# Seroprevalence to the Antigens of *Taenia solium* Cysticercosis among Residents of Three Villages in Burkina Faso: A Cross-Sectional Study

**DOI:** 10.1371/journal.pntd.0000555

**Published:** 2009-11-24

**Authors:** Hélène Carabin, Athanase Millogo, Nicolas Praet, Sennen Hounton, Zékiba Tarnagda, Rasmané Ganaba, Pierre Dorny, Pascal Nitiéma, Linda D. Cowan, Évaluation du Fardeau Économique de la Cysticercose Au Burkina Faso (ÉFÉCAB)

**Affiliations:** 1 Department of Biostatistics and Epidemiology, College of Public Health, University of Oklahoma Health Sciences Center, Oklahoma City, Oklahoma, United States of America; 2 Centre Hospitaltier Universitaire Souro Sanou, Bobo-Dioulasso, Burkina Faso; 3 Institute of Tropical Medicine, Antwerp, Belgium; 4 World Health Organization-Multi Disease Surveillance Centre, Ouagadougou, Burkina Faso; 5 Institut de Recherche en Sciences de la Santé, Bobo-Dioulasso, Burkina Faso; 6 Groupe de Recherche, d'Expertise et de Formation en Santé pour le Développement (GREFSaD), Bobo Dioulasso, Burkina Faso; Universidad Nacional Autónoma de México, Mexico

## Abstract

**Background:**

There is limited published information on the prevalence of human cysticercosis in West Africa. The aim of this pilot study was to estimate the prevalence of *Taenia solium* cysticercosis antigens in residents of three villages in Burkina Faso.

**Methods/Principal Findings:**

Three villages were selected: The village of Batondo, selected to represent villages where pigs are allowed to roam freely; the village of Pabré, selected to represent villages where pigs are usually confined; and the village of Nyonyogo, selected because of a high proportion of Muslims and limited pig farming. Clustered random sampling was used to select the participants. All participants were asked to answer an interview questionnaire on socio-demographic characteristics and to provide a blood sample. The sera were analysed using an AgELISA. The prevalence of “strong” seropositive results to the presence of antigens of the larval stages of *T. solium* was estimated as 10.3% (95%CI: 7.1%–14.3%), 1.4% (0.4%–3.5%) and 0.0% (0.0%–2.1%) in the 763 participants who provided a blood sample in Batondo, Pabré and Nyonyogo, respectively. The prevalence of “weak” seropositive test results to the presence of antigens of the larval stages of *T. solium* was 1.3% (0.3%–3.2%), 0.3% (0.0%–1.9%) and 4.5% (2.0%–8.8%) in Batondo, Pabré and Nyonyogo, respectively. The multivariate logistic regression, which included only Batondo and Pabré, showed that village, gender, and pork consumption history were associated with AgELISA seroprevalence.

**Conclusions/Significance:**

This study illustrates two major points: 1) there can be large variation in the prevalence of human seropositivity to the presence of the larval stages of *T. solium* cysticercosis among rural areas of the same country, and 2) the serological level of the antigen, not just whether it is positive or negative, must be considered when assessing prevalence of human cysticercosis antigens.

## Author Summary

*Taenia solium* cysticercosis is a neglected tropical zoonosis transmitted between humans and pigs. This infection is particularly prevalent in areas where sanitation, hygiene and pig management practices are poor. There is very little information about the importance of this infection in West Africa, even though pork meat is widely consumed in many areas. This pilot study, conducted in three villages of Burkina Faso, demonstrated that people living in areas where pigs are raised were more likely to be infected with cysticercosis than people living in a Muslim village in which there were very few pigs. It also demonstrated variation in the level of infection between the two villages where pigs were raised. Finally, the results suggest that the source of infection in these three villages may differ. These results are significant because they show that there is clustering of infection within villages, even if they are geographically very close to one another. This should encourage future researchers not to combine data from several villages into one summary value. In addition, more work is needed to better describe different potential sources of infection among villages.

## Introduction

*Taenia solium* is a tapeworm transmitted among humans and between humans and pigs. Taeniasis is acquired by humans when eating raw or undercooked pork contaminated with cysticerci, the larval stage of *T. solium*. When ingested, the cysticerci migrate to the intestine of humans where they establish and become adults. These adult worms shed eggs in human feces that can infect other humans and pigs by direct contact or by indirect contamination of water or food. This can be especially problematic in developing countries where pigs are often allowed to roam freely and to eat human feces and where levels of sanitation and hygiene are poor. Ingested eggs result in larval worms which migrate to different parts of the pig or human body and form cysts. A principle site of establishment of the larvae in humans is the central nervous system. Human neurocysticercosis (NCC) occurs when the cysts develop in the brain or spinal cord. Seizures are believed to be the most common presentation of NCC, affecting from 66% to 90% at some stage of their disease [1],[2].

There is limited published information on the prevalence of human cysticercosis in West Africa. In Burkina Faso, no prevalence study has ever been conducted, although NCC has been reported. In a retrospective review of the medical records of 532 persons with seizure disorder seen either as inpatients or outpatients at Yalgado Ouédraogo Teaching Hospital in Ouagadougou, 6.3% of the 158 cases in whom a presumed cause was identified were attributed to NCC based on clinical evidence [3]. No imaging was used to confirm the diagnosis which could have lead to an underestimation of the proportion of seizure cases attributable to NCC. In addition, no definition of “clinical evidence of cysticercosis” was provided, whichmakes this estimate very difficult to interpret. Case reports of human cysticercosis in Burkina Faso have also been published [4]. A third study reviewed 3410 histopathological samples from any location (surgical and biopsy) collected between 1991 and 1995 at the two reference hospitals in Bukina Faso and found 18 with evidence of current infection with *T. solium* larvae [5].

In neighboring pig-raising countries, community-based seroprevalence estimates of cysticercosis in humans range from 1.3% to 3.95% [6]–[9]. There have also been case reports of human cysticercosis in Ivory Coast, Ghana and Senegal [10].

The main objective of the present study is to estimate the prevalence to the antigens of *T. solium* cysticercosis as an indicator of current infection, in three villages in Burkina Faso. A secondary aim is to measure the association between potential risk factors and the prevalence of seropositivity to the antigens of *T. solium* larval stages.

## Methods

### Ethics statement

Informed consents for the interviews of participants and the provision of blood samples were obtained separately. The consent process was done orally because a very large proportion of the population had never been to school (62.5%). Oral consent was documented on the individual consent forms by the research staff. The study protocol was reviewed and approved by the ethical committee of the Center MURAZ (Ref. 02-2006/CE-CM) and by the Institutional Review Board of the University of Oklahoma Health Sciences Center (IRB# 12694) in regard to both human and porcine participants. Both IRBs approved the use of oral consents. The sampling of blood from pigs was approved by the OUHSC IACUC committee (approval #06-018).

### Study sites

**Figure 1:**
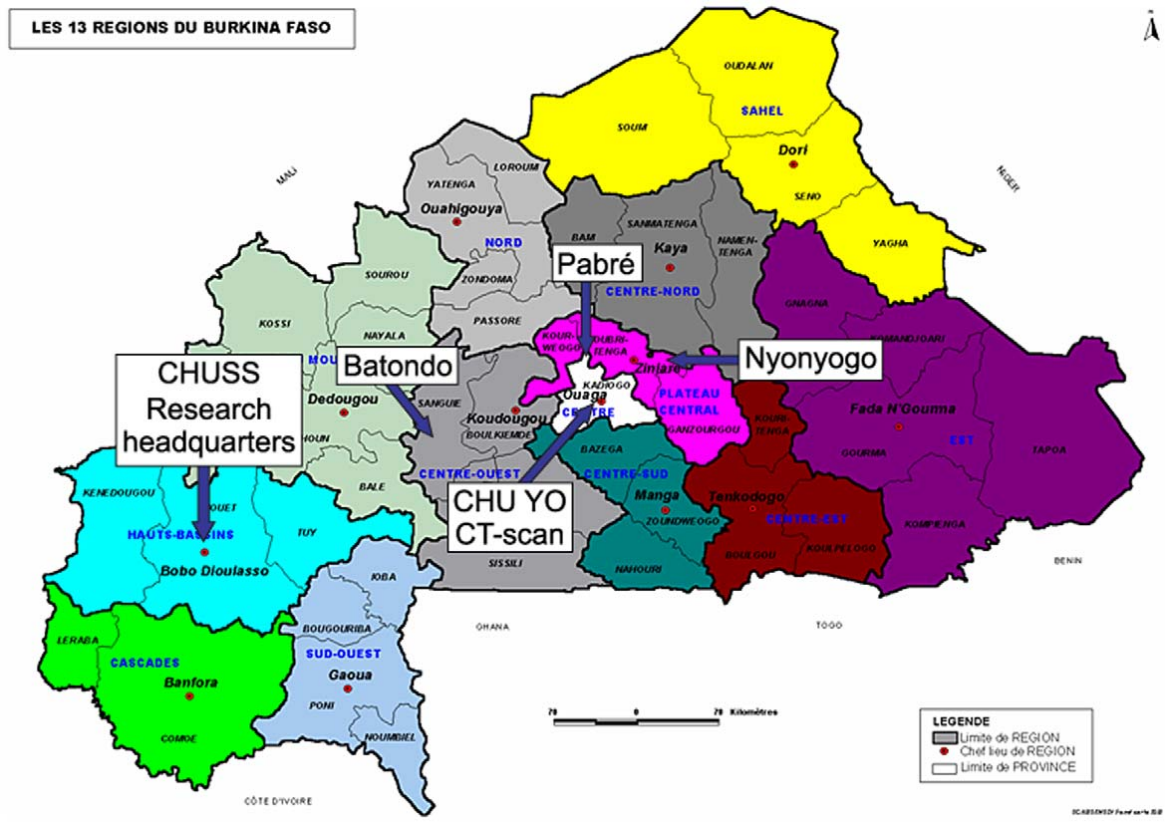
Location of the 3 pilot villages, the research headquarters and of the CT-scan facility.

The pilot study was conducted in the villages of Batondo, Pabré and Nyonyogo, located close to the Capital City of Ouagadougou (Figure 1). The three villages were conveniently selected to represent three types of pig managements. The village of Batondo, located in the commune of Ténado (province of Sanguié) 140 km west of Ouagadougou, was selected to represent villages where pigs are owned and raised by women and are allowed to roam freely. The village of Pabré, in the commune of Pabré (province of Kadiogo), located 25 km north of Ouagadougou, was selected to represent villages where pigs are raised and are usually confined for some period of time during the year. The village of Nyonyogo, located in the commune of Dapelogo (province of Oubritenga) was selected due to a high proportion of Muslims and hence limited pig farming.

### Sampling strategy

A census of all concessions (a grouping of several households usually members of the same family) and households in each village was first conducted. In Batondo and Nyonyogo, all concessions were included. In Pabré, 50% of the concessions were selected at random. Within each concession, all households were included and one person was randomly sampled from each household for participation in the interview and venipuncture for collection of blood samples for serological testing. The random selection was done by placing the names of each household member in a bowl and by asking a child to pick one name from the bowl.

### Study design

This cross-sectional study was conducted between May and October 2007.

### Questionnaire interviews

The head of each household was first interviewed to collect information about each member of his family. In households where pigs were raised, the caretaker of the pigs was interviewed regarding pig management practices. A cooking practices interview was conducted with the wife of monogamous households and with the “senior” woman in polygamous households. The individual sampled at random in each household was asked to answer an epilepsy screening questionnaire which also included socio-demographic information and was administered by a trained member of the study staff. All questionnaires were translated from French to the local languages and back-translated to French. The questionnaires were also pilot tested among a small group of people with and without epilepsy prior to the start of the field study.

### Serological test

Blood samples were left to decant at the end of each sampling day and the sera were put in freezers (−20°C) until the samples were brought to the IRSS (*Institut de Recherche en Sciences de la Santé*) in Bobo-Dioulasso where they were centrifuged and the sera kept at −20°C. The serum samples were tested for circulating antigens of the metacestode of *T. solium* using the enzyme-linked immunosorbent assay (ELISA) [11],[12]. This test is designed to measure the presence of current infection with the larval stages of *T. solium* and not the history of past or present exposure. A seropositive result is indicative of current infection and may or may not be associated with symptoms. The cut-off value was calculated as described by Dorny et al., 2004 [13]. A ratio for each test was calculated dividing the optical density of the sample by the cut-off value. The ratios were used to classify the results as negative (ratio between 0 and 1.0), “weak” positive (ratio between 1.0 and 1.35) and “strong” positive (ratio>1.35). Samples with a coefficient of variation of more than 50% were considered as missing values (n = 3). The sensitivity and specificity of the AgELISA for current infection with cysticercosis has only been reported from a preliminary study conducted in Vietnam. There, the study indicated a sensitivity of 94.4% and a specificity of 100% for the diagnosis of current infection with cysticercosis [14]. The sensitivity and specificity of Ag-ELISA for current cysticercosis infection has been determined in pigs, with a sensitivity and specificity of 86.7% and 96.7%, respectively in Zambia [13], and 76.3% and 84.1%, respectively in South Africa [15].

### Statistical analyses

The prevalence of seropositivity to the larval stages of *T. solium* cysticercosis was estimated separately for “weak” and “strong” seropositivity as the number with “weak” and “strong” positive AgELISA results, respectively, divided by the number of people who provided a blood sample. We then fitted two random-effect models (xtlogit) with the “weak” results assumed either positive or negative to estimate the proportion of the total variance contributed by the village-level clustering (statistic rho).

The results from Nyonyogo were analysed separately and included only in the univariate analyses due to the small number of seropositives and the very small number of pigs raised in that village, which made this village very different from the two others.

For risk factor analyses in Pabré and Batondo, only those with “strong” responses were considered as positive. Univariate associations between being positive to AgELISA and socio-demographic and pork consumption variables at the individual level, as well as pork preparation and pig management variables at the household level were first assessed. Comparisons were made by calculating a prevalence proportion ratio (PPR) with 95% confidence intervals (95%CI). Variables with significant or borderline significant associations with seropositivity in the univariate analyses were then included in a multivariate logistic model adjusting for the effect of village. The results are reported as prevalence odds ratios (POR) with 95% confidence intervals (95%CI). A random-effect logistic regression model with clustering at the concession level was also fitted to take into consideration the clustered nature of the sampling. The results of this model were identical to those from the simple model, however, and thus, only the latter are presented. All analyses were conducted in Stata 10 SE.

## Results

A total of 888 individual interviews were conducted with participants in the three villages. All sampled individuals agreed to answer the interview questionnaire. Of these, 766 (86.3%) provided a blood sample. Table 1 shows the proportion of participants providing a blood sample by selected socio-demographic characteristics. Briefly, the proportion of people providing a blood sample varied somewhat from village to village due to a variety of factors such as the presence of visible veins, the difficulty of obtaining a blood sample, and refusal to provide a sample following the interview. Among those who provided a blood sample, 763 had a valid AgELISA test result.

**Table 1:** Comparison of the socio-demographic characteristics of the participants who provided and did not provide a blood sample for the serological analysis in three pilot villages studied between May and October 2007, Burkina Faso.

Variable		Provided a blood sample	
		Yes N(%)	No N(%)
Number of participants		766 (86.3)	122 (13.7)
Village	Batondo	302 (88.1)	41 (11.9)
	Pabré	288 (80.5)	70 (19.6)
	Nyonyogo	176 (94.1)	11 (5.9)
Sex	Male	372 (88.2)	51 (11.9)
	Female	394 (84.6)	72 (15.5)
Age group	Children (<16)	157 (87.2)	23 (12.8)
	Adult (16–<40)	390 (86.3)	62 (13.7)
	Elderly (≥40)	219 (85.6)	37 (14.5)
Any schooling	Yes	298 (89.5)	35 (10.5)
	No	467 (84.3)	87 (15.7)
Pork eating history*	Never ate pork	247 (90.5)	26 (9.5)
	Ate pork before, not anymore	21 (77.8)	6 (22.2)
	Eats pork now	496 (84.6)	90 (15.4)
Reported having seizures or epilepsy	Yes	60 (89.6)	7 (10.4)
	No	706 (86.0)	115 (14.0)
Reported using the latrine to defecate	Yes	161 (88.5)	21 (11.5)
	No	605 (85.7)	101 (14.3)
Declared ever having had sub-cutaneous nodules**	Yes	16 (2.1)	1 (0.8)
	No	745 (97.9)	125 (99.2)
Ever had tapeworm	Yes	93 (91.2)	9 (8.8)
	No	673 (85.6)	113 (14.4)

*Pork eating history: this variable has 2 missing values.

**Ever had sub-cutaneous nodules: this variable has one missing value.

The prevalence of “strong” seropositive test results was estimated as 10.3% (95%CI: 7.1%–14.3%), 1.4% (0.4%–3.5%) and 0.0% (0.0%–2.1%) in Batondo, Pabré and Nyonyogo, respectively. The prevalence of “weak” seropositive test results was 1.3% (0.3%–3.2%), 0.3% (0.0%–1.9%) and 4.5% (2.0%–8.8%) in Batondo, Pabré and Nyonyogo, respectively. The random-effect models showed that the variance due to the village contributed to a large proportion of the overall variance. The rho statistic was estimated to 0.49 (95%CI: 0.08–0.92) when the “weak” seropositives were assumed negative and to 0.16 (95%CI: 0.03–0.55) when the “weak” seropositives were assumed positive.

Table 2 shows the prevalence of AgELISA seropositivity within categories of several potential risk factors and stratified by village. In Nyonyogo, univariate analyses showed males to have an increased prevalence of presenting a “weak” serological results as compared to females (PPR = 8.02 (95%CI: 1.01, 63.86)). None of the 8 cases with “weak” results had gone to school and none reported using the toilet to defecate. However, only 18.2% and 11.3% of the population of the village had ever attended school and reported using a toilet to defecate, respectively. Nyonyongo children aged less than 16 tended to have a higher prevalence proportion, based on “weak” results, than adults with a PPR = 3.63 (95%CI: 0.95–13.95), and there was a tendency for a higher prevalence in concessions of larger sizes. This latter trend by concession size was observed to a lesser extend in Batondo, but not in Pabré. Except for the association with gender, none of the variables noted in relation to seroprevalence (“weak” only) in Nyonyongo were observed in the other two villages (with “strong” seropositive tests) (Table 2). Because there were so few pigs being raised in Nyonyogo, “weak” seropositive tests were not found to be associated with pork consumption or pig raising in this village.

**Table 2:** Prevalence proportion (%) and (numerator/denominator) of sero-positivity to the AgELISA by several potential risk factors and stratified by village.

Variable	Values	Village		
		Batondo*	Pabré*	Nyonyogo**
Ag-ELISA	“Strong” positive	10.3 (31/300)	1.4 (4/287)	0.0 (0/176)
	“Weak” positive	1.3 (4/300)	0.4 (1/287)	4.6 (8/176)
Schooling	No schooling	10.8 (22/203)	0.9 (1/116)	5.6 (8/143)
	Some schooling	9.7 (9/93)	1.8 (3/170)	0.0 (0/32)
Pork consumption	Never ate pork	2.1 (1/47)	0.0 (0/34)	4.9 (8/164)
	Eats pork medium cooked	15.0 (9/60)	0.0 (0/30)	0.0 (0/2)
	Eats pork well cooked	9.9 (17/171)	1.8 (4/222)	0.0 (0/7)
	Used to eat pork, not anymore	22.2 (4/18)	NO§	0.0 (0/2)
Household size	<5 persons	10.7 (9/84)	2.6 (3/116)	2.4 (1/42)
	5–9 persons	8.2 (10/122)	0.0 (0/148)	4.8 (4/83)
	≥15 persons	13.3 (12/90)	4.6 (1/22)	5.9 (3/51)
Concession size	≤6 persons	7.7 (2/26)	2.0 (3/150)	0.0 (0/44)
	7–15 persons	9.6 (5/52)	0.9 (1/113)	4.4 (3/68)
	≥16 person	11.0 (24/218)	0.0 (0/23)	7.8 (5/64)
Age group	7–15	10.4 (5/48)	1.4 (1/71)	10.5 (4/38)
	16–39	9.4 (15/160)	0.8 (1/131)	3.2 (3/93)
	≥40	12.5 (11/88)	2.4 (2/84)	2.2 (1/45)
Sex	Male	13.6 (20/147)	2.9 (4/139)	8.5 (7/82)
	Female	7.4 (11/149)	0.0 (0/147)	1.1 (1/94)
Ever had taeniasis	Yes	8.8 (3/34)	2.6 (1/38)	1/20 (5.0)
	No	10.7 (28/262)	1.2 (3/248)	7/156 (4.5)
Reported using the latrine to defecate	Yes	4.0 (1/25)	1.8 (2/114)	0.0 (0/20)
	No	11.1 (30/271)	1.2 (2/172)	5.1 (8/156)
Source of drinking water§	Tap or spring or bore well	12.9 (11/85)	0.8 (1/120)	4.8 (8/166)
	Cemented well	5.9 (1/17)	0.0 (0/4)	NO
	Open or traditional well	10.0 (19/191)	1.9 (3/162)	0.0 (0/10)
Number of pigs owned by the household	None	10.1 (10/99)	2.3 (3/129)	4.7 (8/171)
	1–2	12.3 (14/114)	1.6 (1/63)	0.0 (0/3)
	≥3	8.4 (7/83)	0.0 (0/94)	0.0 (0/2)
AgELISA results in pigs owned by the household	No pigs	10.3 (10/97)	2.4 (3/127)	4.7 (8/171)
	“Strong”	8.3 (4/48)	0.0 (0/51)	NO
	“Weak”	25.0 (1/4)	0.0 (0/3)	NO
	Negative	12.9 (11/85)	0.0 (0/68)	0 (0/5)
	No test conducted	8.1 (5/62)	2.7 (1/37)	NO

*Batondo and Pabré: only strong positive responses reported.

**Nyonyogo: all responses were weak positives.

**§:** Source of drinking water: 4 missing observations.

NO = No Observations.

The multivariate logistic regression, which included only Batondo and Pabré, showed that village, gender, and pork consumption habits were associated with “strong” AgELISA seropositivity (Table 3). The odds of being seropostive were considerably higher in Batondo than in Pabré (POR = 8.86; 95%CI = 3.01, 26.14), in men compared to women (POR = 2.34; 95%CI = 1.10, 4.97) and in those eating pork either in the past (POR = 19.62; 95%CI = 1.91, 2010.95) or currently (POR = 8.75; 95%CI = 1.11, 68.88) compared to those who never ate pork. The ownership of pigs by one household member confounded the association between seropositivity and pork consumption such that when this variable was included in the model the association between eating pork and seroprevalence became significant. Although not itself statistically significant, because of this interaction the variable “pig ownership” was retained in the multivariate model.

**Table 3:** Prevalence Odds Ratio (POR) estimates (and 95% Confidence Intervals (95% CI)) from a multivariate logistic regression model adjusting for the village to explore the association between potential risk factors and sero-positivity to the Ag-ELISA test.

Variable	Effect	Reference	OR (95% CI)
Village	Batondo	Pabré	8.86 (3.01, 26.14)
Sex	Male	Female	2.34 (1.10, 4.97)
Pigs raised by a household member	Yes	No	0.53 (0.25, 1.14)
Pork consumption	Ate pork in the past, not now	Never ate pork	19.62 (1.91, 201.95)
	Eats pork now	Never ate pork	8.75 (1.11, 68.88)

## Discussion

This study is the first to estimate the seroprevalence to the presence of antigens to *T. solium* cysticercosis in Burkina Faso. The strengths of this study are that the results are based on a clustered-random sample of residents of three rural villages, the participation proportion for the interview was excellent (100%) and very good (86.3%) for the serology, the participants answered almost all questions in the interviews (few missing values), and the majority of serological tests conducted had valid results.

Our results show considerable variation in seroprevalence in the three study villages. The prevalence to the presence of antigens of *T. solium* cysts was nearly 8 times higher in Batondo than in Pabré. Several reasons may explain the difference between Batondo and Pabré. For example, the proportion of participants who had gone to school was much higher in Pabré (55.6%) than in Batondo (30.0%), and a larger proportion of the participants used the toilet to defecate in Pabré (37.6%) than in Batondo (7.9%). Pigs were also more often penned in Pabré (54.8%) during the rainy season as compared to Batondo (10.9%) where pigs were more often tethered (94.0%). Tethered pigs are more likely than penned pigs to have access to human feces as they are often moved to be able to feed, and hence, are more likely to be exposed to a contaminated site. It was also observed during the field study that pigs were living in closer contact with humans in Batondo than in Pabré. These factors, possibly in addition to other village-level variables that were not measured, may contribute to the lower prevalence of the presence of antigens of *T. solium* cysts in Pabré compared to Batondo.

All seropositive results in Nyonyogo were “weak”. One could speculate, as an hypothesis yet to be tested, that the force of infection from the environment is lower in Nyonyogo due to the presence of very few pigs and therefore, to a lower prevalence of taeniasis among the population. It is also possible that the source of infection is different in Nyonyogo compared to that in the other villages as discussed below. Another hypothesis is that most of the infections in Nyonyogo were either very recent or old resulting in a lower density of antigens in the blood.

Although based on a small number of “weak” cases, the finding that children tended to have a higher seroprevalence than adults only in Nyonyogo deserves further exploration. One hypothetical explanation for this observation is that children in Nyonyogo acquire infection through playing in the contaminated environment since few adults consume pork and thus are at lower risk for taeniasis. Sanitation was very poor in Nyonyogo with only 14.4% of the household having a toilet and 11.2% of the people using the latrine to defecate. In the other two villages, more people consume pork and the prevalence of taeniasis is probably higher, which could increase the risk of auto-infections or infection through the contamination of food and water. This possibility could also explain the difference in the strength of AgELISA optical densities between the villages. These interpretations are hypotheses which would need to be verified in a larger cohort study.

To our knowledge, there has been only one other community-based sero-survey done in Sub-Saharan Africa on the presence of antigens to the larval stages of *T. solium* cysticercosis [9], which reported a seroprevalence varying between 0.4% and 3.0% in the three rural communities in Menoua district, Cameroon, between 1999 and 2000. One important limitation of this study is that sampling was done among volunteers. Nonetheless, the study's results indicate some variation in prevalence by community, but less variation than what was observed in the present study in which participants were selected according to a clustered random sampling strategy. Also, we had specifically sampled a village where the majority of the population was Muslim and where there were very few pigs being raised.

In a recent hospital-based case-control study from the Kiremba area of Burundi, the prevalence of seropositivity using AgELISA in controls (persons without epilepsy) was estimated as 20% [16]. In this study, 80% of the controls were people being vaccinated at the Kiremba area hospital and age-matched to a group of patients with epilepsy who were being seen at that hospital. This design makes it difficult to assess the source of the controls and therefore assess to what extent they represent the general population since controls were age matched to epilepsy patients. In a study in Cameroon of people with epilepsy receiving care in rural clinics, the seroprevalence by AgELISA was estimated as 1.2% [17]. The estimated prevalence of seropositivity to the antigens of *Taenia solium* cysts among people in our sample with confirmed epilepsy was considerably higher at 15.2%.

In the Burundi case-control study, the POR of seropositivity to the antigens of *Taenia solium* cysts was 2.5 (95%CI = 1.8, 3.4) when comparing males to females and 1.7 (95%CI: 1.1–2.5) when people eating pork were compared to those not eating pork [16], results similar to those of the present study, although the association with pork consumption in our cross-sectional study was much larger.

In Batondo and Pabré, the presence of pigs within the household tended to reduce the seroprevalence and it confounded the association between pork consumption and seroprevalence. This is because there was a higher proportion of participants from households where pigs were raised who consumed pork than in household where pigs were not raised. This uneven distribution of pork consumption according to the presence of pig raising leads to an underestimation of the effect of pork consumption on the seroprevalence to the antigens of *Taenia solium* cysts if not adjusted. The proportion of participants aware of the link between consumption of undercooked pork and taeniasis was similar among people who raised pigs (15.1%) and those who did not (14.2%). It may be that members of households where pigs are not raised are more likely to consume pork at the market than are those from household where pigs are raised, which may expose them to greater risk than those eating their meals at home. Unfortunately, our questionnaire did not include a question on the location of consumption of pork meat, which would have helped in explaining this confounding factor.

This study has some limitations. First, 13.7% of the 888 interviewed participants did not provide a blood sample. The reasons for not providing a sample may have been linked to the difficulty in obtaining blood at the time of sampling or refusal to provide a sample following the interview. Refusals were a minority of those who did not provide a sample. Due to this, we believe that important selection bias is unlikely. Second, it would have been very interesting to re-test participants with “weak” results a few months later. This would have indicated whether those “weak” cases were new or old infections. Unfortunately, this was not feasible in the context of this project. Third, it would have also been interesting to obtain results from a valid antibody serological test. We did test the samples for the presence of antibodies according to the method of Arruda et al. 2005 [18]. However, this test is now thought to be invalid due to several cross reactions with other helminthes and protozoa such as Echinococcus, Filaria , Fasciola , Strongyloides, Schistosoma, Toxocara, Amoeba ,and Plasmodium [19]. Another alternative would have been to test the samples using the well validated Western Blot test (EITB) for the detection of antibodies to *T. solium* [20]. This was not feasible due to the complexity and cost of the test. However, as a test for the presence of current infection with the larval stages of *T. solium*, preliminary studies indicated a sensitivity of 94.4% and a specificity of 100% of the Ag ELISA test in Vietnam [14].

We have shown that the prevalence to the antigens of the larval stages of *T. solium* can be very high in some villages of Burkina Faso and virtually nonexistent in other villages in the same region. This study illustrates two major points; first, there can be large variation in the prevalence of antigens to human cysticercosis among rural areas of the same country, and second, the serological level of the antigen, not just whether it is positive or negative, must be considered when analyzing data in order to arrive at more valid conclusions. The first point is especially relevant to studies of the burden of diseases. If all studies are concentrated in areas where pigs are roaming, the overall burden of the infection for the country would be over-estimated; the converse would be true as well.

## Supporting Information

Checklist S1 STROBE checklist.
(0.08 MB DOC)
